# BMI-1 expression increases in oral leukoplakias and correlates with cell proliferation

**DOI:** 10.1590/1678-7757-2019-0532

**Published:** 2020-04-27

**Authors:** Isadora Peres KLEIN, Luise MEURER, Chris Krebs DANILEVICZ, Cristiane Helena SQUARIZE, Manoela Domingues MARTINS, Vinicius Coelho CARRARD

**Affiliations:** 1 Universidade Federal do Rio Grande do Sul Faculdade de Odontologia Departamento de Odontologia Conservadora Porto AlegreRio Grande do Sul Brasil Universidade Federal do Rio Grande do Sul, Faculdade de Odontologia, Departamento de Odontologia Conservadora, Porto Alegre, Rio Grande do Sul, Brasil.; 2 Universidade Federal do Rio Grande do Sul Faculdade de Medicina Departamento de Patologia Porto AlegreRio Grande do Sul Brasil Universidade Federal do Rio Grande do Sul, Faculdade de Medicina, Departamento de Patologia, Porto Alegre, Rio Grande do Sul, Brasil.; 3 University of Michigan Department of Periodontics and Oral Medicine Laboratory of Epithelial Biology Ann ArborMichigan United States of America University of Michigan, Department of Periodontics and Oral Medicine, Laboratory of Epithelial Biology, Ann Arbor, Michigan, United States of America.

**Keywords:** Leukoplakia, oral, Clinical evolution, Carcinoma, squamous cell

## Abstract

Oral leukoplakia (OL) is a white lesion of an indeterminate risk not related to any excluded (other) known diseases or disorders that carry no increased risk for cancer. Many biological markers have been used in an attempt to predict malignant transformation; however, no reliable markers have been established so far.

**Objective:**

To evaluate cell proliferation and immortalization in OL, comparing non-dysplastic (Non-dys OL) and dysplastic OL (Dys OL).

**Methodology:**

This is a cross-sectional observational study. Paraffin-embedded tissue blocks of 28 specimens of Non-dys OL, 33 of Dys OL, 9 of normal oral mucosa (NOM), 17 of inflammatory hyperplasia (IH), and 19 of oral squamous cell carcinomas (OSCC) were stained for Ki-67 and BMI-1 using immunohistochemistry.

**Results:**

A gradual increase in BMI-1 and K-i67 expression was found in oral carcinogenesis. The immunolabeling for those markers was higher in OSCC when compared with the other groups (Kruskal-Wallis, p<0.05). Ki-67 expression percentage was higher in OL and in IH when compared with NOM (Kruskal-Wallis/Dunn, p<0.05). Increased expression of BMI-1 was also observed in OL when compared with NOM (Kruskal-Wallis/Dunn, p<0.05). No differences were observed in expression of both markers when non-dysplastic and dysplastic leukoplakias were compared. A significant positive correlation between Ki-67 and BMI-1 was found (Spearman correlation coefficient, R=0.26, p=0.01). High-grade epithelial dysplasia was associated with malignant transformation (Chi-squared, p=0.03).

**Conclusions:**

These findings indicate that BMI-1 expression increases in early oral carcinogenesis and is possibly associated with the occurrence of dysplastic changes. Furthermore, our findings indicate that both Ki-67 and BMI-1 are directly correlated and play a role in initiation and progression of OSCC.

## Introduction

Oral leukoplakia (OL) is a lesion with a risk of malignant transformation ranging from 0.13% to 17.5%.^1^ Histopathologically, OL is characterized by a variety of epithelial changes, including dysplasia. The presence of epithelial dysplasia is considered the most important predictive factor for OL prognosis.^1 - 3^

Although many biological markers have been explored, no reliable ones have yet been established for predicting malignant transformation of OL, thus necessitating additional studies to increase our knowledge of the biological processes underlying OL and oral carcinogenesis.^4^ Ki-67 is a nuclear protein expressed in the G1, S, G2, and M phases of the cell cycle, and its expression reflects the total growth fraction in tissues.^5^ Ki-67 expression correlates with the severity of epithelial dysplasia and histological grading of oral squamous cell carcinoma (OSCC).^6 , 7^ Another protein named BMI-1, which mediates gene silencing by regulating chromatin structure,^8 , 9^ plays a central role in cell cycle regulation and cell immortalization, as well as cell senescence and epithelial-mesenchymal transition (EMT).^10 , 11^ BMI-1 is associated with the initiation and progression of various tumors, including oropharyngeal,^10^ nasopharyngeal,^11^ and prostate^12^ cancers. Furthermore, increased BMI-1 expression was found in bronchial premalignant lesions as well as squamous cell carcinoma (SCC), indicating that its expression in neoplastic cells may be an early event in lung carcinogenesis.^13^ BMI-1 is also overexpressed in OSCC cells when compared with normal oral mucosa cells and has been presumed to influence proliferation and immortalization of epithelial cells in oral carcinogenesis.^14^

This study aimed to evaluate cell proliferation and immortalization in OL by studying Ki-67 and BMI-1 expression in non-dysplastic (Non-dysOL) and dysplastic (DysOL) cases.

## Methodology

### Patients and tissue specimens

Ninety-eight cases of OL reported between 2000 and 2014 were selected for this study. Tissue samples were obtained from the archives of the Laboratories of Pathology of the Hospital de Clínicas de Porto Alegre and the School of Dentistry of the Federal University of Rio Grande do Sul considering the clinical impression of leukoplakia. The study protocol was approved by Human Research Ethics Committee (CAAE 26759114900005327).

**Figure 1:**
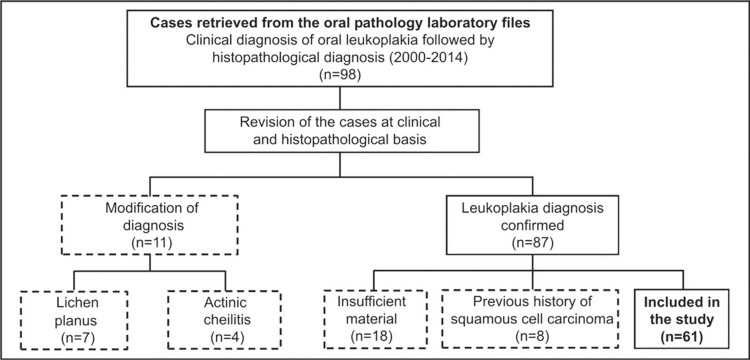
Flowchart of sampling strategy, depicting the criteria to select the study sample. After revision, the cases in which oral leukoplakia was confirmed were subjected to a strict evaluation to define if the amount of tissue was enough to prepare the required number of histological sections. Cases with previous history of squamous cell carcinoma were discarded

Information on demographics, risk factors, clinical presentation, treatment, and prognosis are shown in Table 1 . Moreover, histological slides were revised to exclude cases incompatible with OL and cases with incomplete information in medical records or insufficient material for sampling. Descriptive histopathological diagnosis such as atrophy, acanthosis, epithelial hyperplasia, hyperkeratosis, and epithelial dysplasia were considered microscopic features of a clinical diagnosis of leukoplakia. After selection, the medical records were evaluated to confirm the clinical hypothesis and the final diagnosis of leukoplakia after clinical and microscopic correlation. Cases of *in situ* carcinoma were excluded. After that, 61 cases of OL were included for analysis ( Figure 1 ). Nine cases of normal oral mucosa (NOM) obtained during surgical removal of unerupted third molars and, 17 cases of inflammatory hyperplasia (IH), and 19 cases of OSCC were used as comparison groups. IH cases were included as a reference for benign lesions that show an increased cell proliferation without the potential for malignant transformation.

**Table 1:** Demographic and clinical characteristics of the OL and OSCC patient

	OL	OSCC	p
CHARACTERISTIC			
All patients, no.(%)	61 (76.25)	19 (23.75)	
**Age, years**			
Mean	58.0	59.4	0.65*
Standard deviation	12.8	10.9	
Range (minimum-maximum)	26-81	39-82	
**Gender, no(%)**			
Female	26 (42.6)	6 (31.6%)	0.56**
Male	35 (57.4)	13 (68.4%)	
**Tobacco habits, no(%)**			
Never	8 (16.3)	2 (13.3%)	0.71**
Past and present	41 (83.7)	13 (86.7%)	
Unknown	12	4	
**Alcohol consumption, no(%)**			
Never	8 (17.4)	3 (25.0%)	0.68**
Past and present	38 (82.6)	9 (75.0%)	
Unknown	15	7	
**Location, no(%)**			
Tongue / floor of the mouth	19 (31.6)	12 (63.2%)	0.02**
Other locations	41 (68.4)	7 (36.8%)	
Unknown	1	0	
**Clinical type, no(%)**			
Homogeneous	32 (60.4)	_	_
Non homogeneous	21 (39.6)	_	
Unknown	8	_	
**Lesion size, no(%)**			
<2 cm	30 (60.0)	_	_
≥2 cm	20 (40.0)	_	
Unknown	11	_	
**TNM, no(%)**			
I/II	_	5(27.8%)	_
III/IV	_	13(72.2%)	
Unknown	_	1	
**Presence of epithelial dysplasia, no(%)**			
No	28 (45.9)	_	_
Yes	33 (54.1)	_	
**Grade of epithelial dysplasia, no (%)**			
Absent	28 (45.9)	_	_
Low grade	22 (36.1)	_	
High grade	11 (18.0)	_	

*Student’s t test; **Chi-square test

### Clinical and follow up data

Clinical data regarding lesion location, clinical presentation and lesion size were obtained from the clinical forms of patients seen in the School of Dentistry. Patients who did not present new lesions and maintained the clinical characteristics and/or OSCC development during the follow-up period were considered as having good prognosis. Patient who presented new lesions, increased lesion size, changes in surface and/or color, as well as those who developed OSCC were considered as having poor prognosis.

### Histopathological analysis

All selected samples were fixed in 10% neutral formalin and embedded in paraffin. Five-micrometer H&E (hematoxylin and eosin) stained sections were blindly reviewed by two pathologists (V.C.C. and I.P.K.). The epithelial dysplasia in OL samples was graded according to the criteria and definition proposed by Reibel, et al.^15^ (2017) and Kujan, et al.^16^ (2006). A consensus was reached in cases that were graded differently by the two pathologists.

### Immunohistochemistry

Tissue sections were subjected to immunohistochemical staining for BMI-1 and Ki-67 antigens. Shortly after, blocks were sectioned (3 μm) and placed on silanized slides. The slides were subsequently deparaffinized in xylene and hydrated in descending grades of ethanol. Antigen retrieval for Ki-67 was performed for 18 h in low pH solution in a 90°C water bath, and for BMI-1, Tris-HCl buffer (pH 8.5) for 20 min at 98°C was used for the water bath. The slides were then incubated with the primary antibodies: Ki-67 (MIB-1, DAKO, 1:50, 1 h) and BMI-1 (ab14389, Abcam, 1:100, 1 h). The EnVision (DakoCytomation, Carpinteria, CA, USA) was used as the detection system. The sections were then incubated with diaminobenzidine tetrahydrochloride (DAB, Novocastra, Newcastle, UK) and counterstained with Mayer’s hematoxylin. The primary antibody was omitted for the negative control. The human appendix and reactive lymph node tissue were used as positive controls for BMI-1 and Ki-67, respectively. Only nuclear brown staining was considered as positive marking, regardless of the color intensity.^17 , 18^

### Immunostaining evaluation

Images of the selected fields were captured using a conventional light microscope (CX41RF model, Olympus Latin America, Inc., Miami, Florida, USA) coupled to a camera (QColor 5, Coolet, RTV, Olympus Latin America, Inc., Miami, Florida, USA) and connected to a computer (Dimension 5150, Dell, Porto Alegre, RS, Brazil). Images were analyzed using the QCapture software program (Quantitative Imaging Corporation, Inc., Surrey, DC, Canada, version 2.81). Immunohistochemical evaluation was performed under high-power magnification (×400). Nuclear staining was considered for positivity, regardless of staining intensity. Ki-67 were counted and classified based on Gonzalez-Moles, et al.^5^ (2000). The quantitative analysis involved the analysis of images of the slides using the same aforementioned imaging system. The labeling index (LI) was determined by the percentage of labeled nuclei *per* 1000 cells of all specimens of each group.^5^ BMI-1 was analyzed semi-quantitatively using scores based on the percentage of positive cells. To each case was assigned a score as follows: 1 (up to 50% positive cells - low), 2 (50 – 80% - moderate), and 3 (over 80% - high).^19^

### Statistical analysis

The Kruskal-Wallis test, followed by Dunn’s test, was used for multiple comparisons and the Chi-square test was used for comparisons of scores distribution among the groups. Student *t* /ANOVA and Mann-Whitney/Kruskal Wallis tests were used to assess the influence of OL characteristics on Ki-67 and BMI-1 expression, respectively. Spearman’s correlation analysis was conducted to determine the relationship between BMI-1 and Ki-67 expression. The SPSS Statistics software, 18.0 Version, was used for statistical analysis, and p value threshold used was 5%.

## Results

### Demographic and clinical characteristics

Table 1 shows the demographic and clinical characteristics of the OL and OSCC patients. The cases were divided into two groups, namely “tongue or floor of the mouth” and “other sites”, according to the lesion site.^20^ The tongue or floor of the mouth group had a significantly higher percentage of OSCC cases (Student’s t test, p=0.02) when compared with OL cases. Regarding prognosis, twenty-two (55.0%) patients had good prognosis and 18 (45.0%), poor prognosis.

Table 2 shows the characteristics of OL without and with OSCC development during the follow-up period ranging from 12 to 156 months (56.9±33.0). Follow-up information was not available in 21 cases (34.4%), whereas it was available for 40 OL patients (65.5%). Four of the 40 OL patients (10.0%) developed OSCC, resulting in a 2.1% annual malignant transformation rate. Of these 4 patients, 3 (75.0%) patients underwent malignant transformation in the tongue or floor of the mouth and exhibited a high grade dysplasia (Chi-square test, p<0.01).

**Table 2:** Demographic and clinical characteristics of the OL with and without OSCC development

	OL patients without OSCC development	OL patients with OSCC development	p
**Age**			
Mean (SD)	56.3 (13.0)	52.7 (15.9)	0.61*
Min-Max	26-79	31-68	
**Gender**			
Male	23 (63.9)	2 (50.0)	0.62**
Female	13 (36.1)	2 (50.0)	
**Location**			
Tongue/floor of the mouth	11 (30.6)	3 (75.0)	0.11*
Others	25 (69.4)	1 (25.0)	
**Clinical type**			
Homogeneous	20 (64.5)	1 (33.3)	0.54**
Non-homogeneous	11 (35.5)	2 (66.7)	
Unknown	5	1	
**Lesion size**			
<2cm	17 (54.8)	1 (50.0)	1.00**
≥2cm	14 (45.2)	1 (50.0)	
Unknown	5	2	
**Presence of epithelial dysplasia**			
No	17 (47.2)	1 (25.0)	0.01**
Yes	19 (52.8)	3 (75.0)	
**Epithelial dysplasia grade**			
Absent	18 (50.0)	1 (25.0)	0.03**
Low risk	12 (33.3)	0 (0.0)	
High risk	6 (16.7)	3 (75.0)	

*Student’s t test; **Chi-square test

### Immunohistochemical analysis

Ki-67 expression increases in IH, OL and OSCC ( Table 3 ).

**Table 3:** Percentage of immunopositive cells for Ki-67 and BMI-1 in normal oral mucosa (NOM), inflammatory hyperplasia (IH), and oral leukoplakia (OL)

	NOM	IH	Non-dys OL	Dys OL	SCC	p
**Ki-67 immunolabeling**						
Mean	10.2^A^	29.4^B^	33.1^B^	36.3^B^	62.8^C^	<0.01*
SD	3.0	8.9	11.1	15.2	16.6	
**BMI-1 immunolabeling**						
Mean	1.7^A^	2.1^B^	2.4^B^	2.5^B^	2.8^C^	<0.01**
SD	0.5	0.9	0.7	0.7	0.4	

* ANOVA/Tukey, **Kruskal-Wallis test/Dunn. Means followed by different uppercase letters are different from each other

**Figure 2:**
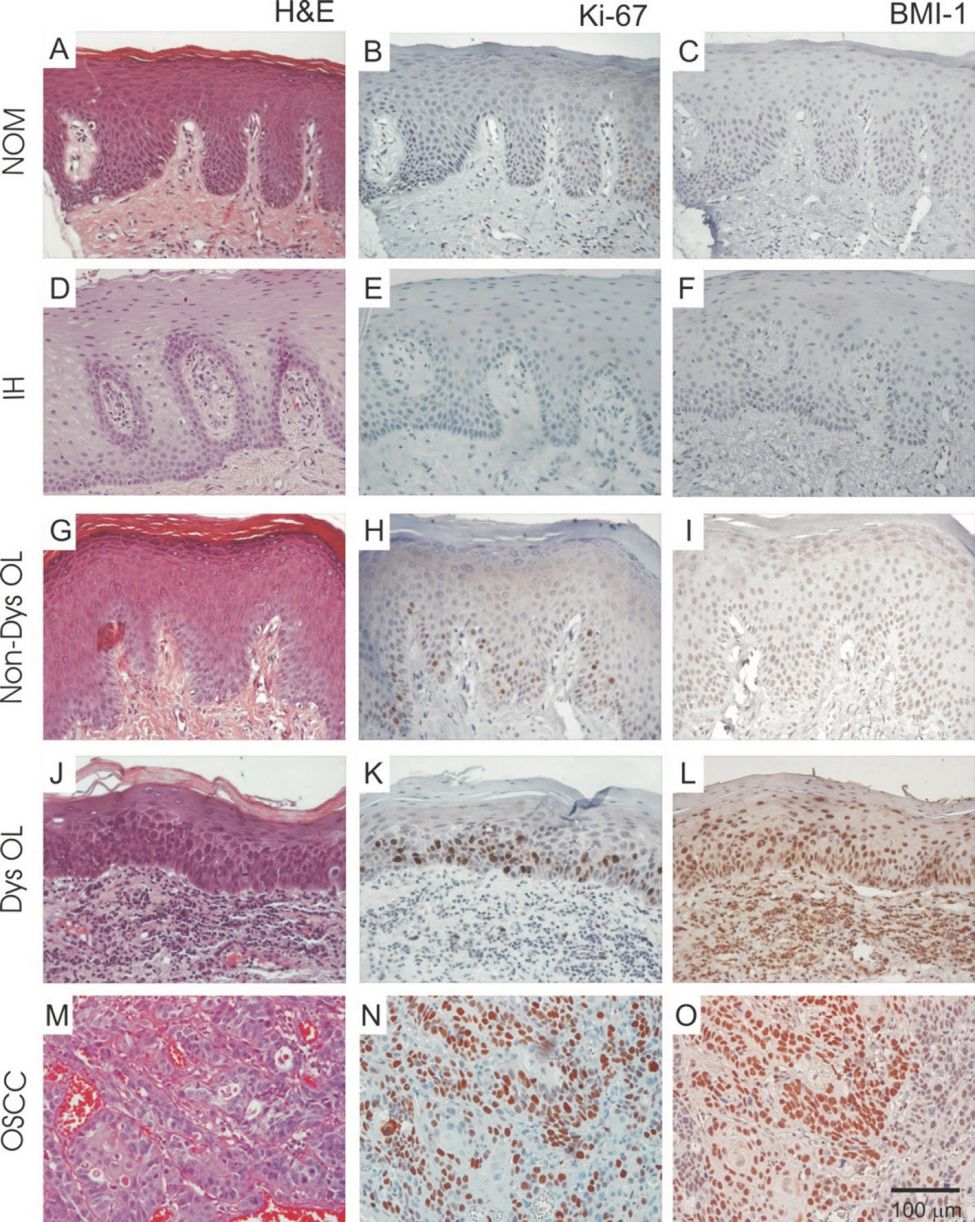
Gradual increase of Ki-67 and BMI-1 expression is observed from to normal oral mucosa (NOM) to oral squamous cell carcinoma (OSCC). Representative photomicrographs of NOM, inflammatory hyperplasia (IH), non-dysplastic oral leukoplakia (Non-dys OL), dysplastic oral leukoplakia (Dys OL) and OSCC. Original magnification ×400

Ki-67 immunolabeling data is shown in Figure 2 . There was a gradual increase in Ki-67 expression (ANOVA/Tukey, p<0.01) through NOM (10.2%), IH (29.4%), Non-dysOL (33.1%), DysOL (36.3%) and OSCC (62.8%). Moreover, cell proliferation was higher (ANOVA/Tukey, p<0.01) through NOM to OSCC. Comparisons among different grades of epithelial dysplasia showed no statistically significant differences (Kruskal Wallis, p>0.05). Representative images of Ki-67 immunolabeling are shown in Figure 2 .

BMI-1 expression increases in IH, OL and OSCC ( Table 3 ).

Increasing positivity was observed for BMI-1 through NOM (1.7%), IH (2.1%), Non-dysOL (2.4%), DysOL (2.5%) and OSCC (2.8%), agreeing with the Ki-67 expression pattern. Non-dysOL cases showed lower BMI-1 immunolabeling when compared with the DysOL cases, although this difference was not statistically significant. However, the means were statistically higher (Kruskal Wallis, p<0.01) in OL when compared with that in NOM, and in OSCC, when compared with that in NOM, IH and OL. Representative images of BMI-1 immunolabeling are shown in Figure 2 .

### Correlation between BMI-1 and Ki-67 expression

Spearman’s correlation coefficients were estimated to determine if BMI-1 expression could be associated with changes in cell proliferation. BMI-1 expression directly correlated with Ki-67 expression (Spearman’s correlation, R=0.26, p=0.01).

### Association of Ki-67 and BMI-1 immunolabeling with OL clinical characteristics and histopathological changes (Table 4)

Higher Ki-67 and BMI-1 expression was observed in lesions in the tongue and floor of the mouth, epithelial dysplasia, OSCC development and poor clinical evolution; however, the associations were not statistically significant.

**Table 4:** Clinical and histopathological characteristics in OL cases and the association with the expression of Ki-67 and BMI-1

	Ki-67(%)		BMI-1 (score)	
	Mean(SD)	p	Mean(SD)	p
**Location**				
Tongue / floor of the mouth	23.1 (12.4)	0.69^a^	2.6 (0.6)	0.42^b^
Other locations	38.3 (11.5)		2.4 (0.7)	
**Clinical type**				
Homogeneous	32.3 (14.5)	0.24^a^	2.5 (0.7)	0.84^b^
Non-homogeneous	27.7 (12.8)		2.5 (0.6)	
**Lesion size**				
<2 cm	24.7 (12.9)	0.39^a^	2.4 (0.7)	0.52^b^
≥2 cm	28.4 (13.3)		2.6 (0.6)	
**Presence of epithelial dysplasia**				
No	33.1 (11.1)	0,58^a^	2.4 (0.7)	0.44^b^
Yes	36.3 (15.2)		2.5 (0.7)	
**Grade of epithelial dysplasia**				
Absent	33.1 (11.1)	0.08^c^	2.4 (0.1)	0.37^d^
Low grade	33.0 (15.7)		2.4 (0.2)	
High grade	43.0 (12.4)		2.7 (0.1)	
**OSCC development**				
No	25.4 (12.6)	0.24^a^	2.3 (0.7)	0.26^b^
Yes	33.5 (14.9)		2.6 (1.0)	
**Clinical evolution**				
Good	24.7 (12.9)	0.39^a^	2.3 (0.6)	0.23^b^
Poor	24.8 (13.3)		2.5 (0.8)	

^a^Student’s t test; ^b^Mann Whitney’s test; ^c^ANOVA; ^d^Kruskal-Wallis

## Discussion

Oral carcinogenesis is a complex process resulting from various genetic and epigenetic changes. BMI-1 overexpression in OSCC cells has been suggested to be associated with an increased cell proliferation^14^ and to be a predictive of tumorigenesis.^21 , 22^ Interestingly, statistically significant changes were observed in early stages of carcinogenesis.^21 , 22^ Moreover, BMI-1 immunolabeling levels were directly associated with cell proliferation in epithelium during carcinogenesis.^23^ To the best of our knowledge, this study is the first to assess Ki-67 and BMI-1 expression in OL.^19^ As expected, the expression of these markers was increased in OL when compared with NOM.

BMI-1 is involved in the transcriptional repression of Hox genes, thus affecting stem cell self-renewal, embryonic development, and proliferation.^24 , 25^ Our results show that BMI-1 expression increases during carcinogenesis. However, BMI-1 immunolabeling analysis showed no differences between OL and IH. OL presented a higher and statistical positivity of BMI-1 when compared with NOM. Our results agrees with those of Kang, et al.^14^ (2007), who reported that increased BMI-1 expression was associated with dysplastic changes during oral carcinogenesis. The role of BMI-1 in EMT demonstrated in previous studies with breast cancer cells further support our findings. Moreover, BMI-1 overexpression in cancer cells has been reported to activate PI3K/AKT signaling pathway, and induce cell migration and metastasis.^26 , 27^

Liu, et al.^28^ (2012) found that BMI-1 expression was associated with the development of oral cancer in patients with OL, suggesting that immunohistochemical marker could be used as a predictor of OL transformation. In that study, approximately 13% of 135 OL patients demonstrating BMI-1 positivity developed OSCC when compared with 10.3% patients negative for BMI-1. In our study, 10.0% of OL patients who were followed-up developed OSCC. In these patients, the presence of high grade dysplasia was found to be a predictor for OSCC development. The same group of patients displayed an increased Ki-67 and BMI-1 expression; however, this was not statistically significant. Regarding the lesion site, three of the four lesions were located in the tongue/floor of the mouth, reinforcing that lesions at these sites present a more aggressive behavior.^20^ The annual malignant transformation rate observed in our study agrees with that reported by other recent studies.^20^ An increased cell proliferation is well known to be one of the main events of carcinogenesis.^29 , 30^ Our findings showed an increased percentage of Ki-67 positive cells in Non-dysOL and DysOL. Moreover, cell proliferation was higher in OSCC when compared with NOM.

Although the immunoexpression of Ki-67 was slightly higher in DysOL than in Non-dysOL, the difference was not statistically significant, as found in previous studies.^30 , 31^ Furthermore, the same study showed that Ki-67 expression was progressively higher depending on the degree of epithelial dysplasia.^32^ Therefore, our findings reinforce that Ki-67 expression is a valuable predictive marker for oral leukoplakia progression, reinforcing the presence of mitosis in the upper half of the epithelium as an important criterion in the morphological analysis, as recommended by previous studies.^6 , 33^

The other important result was the significant correlation found between Ki-67 and BMI-1 immunolabeling. This may be attributed to the role of BMI-1 in the regulation of cell proliferation by suppressing INK4a expression, a locus that triggers senescence in human somatic cells.^34^ Therefore, the switch between differentiation and epithelial-mesenchymal transition, which depends on genetic and epigenetic events, is modulated by the control of cell growth, survival, angiogenesis, and motility. The balance of cross-talk between these signaling pathways is the basis for acquiring a malignant phenotype and progression to OSCC. Despite the large number of studies on this subject, the knowledge about individual factors must be improved to develop strategies for cancer prevention.^35^

To the best of our knowledge, this study is the first to report the correlation between Ki-67 and BMI-1 expression in OL. Based on the results, cell proliferation and changes towards epithelial-to-mesenchymal transition are likely related events during carcinogenesis. The increased expression of BMI-1 in the early stages of development of oral carcinogenesis indicates its potential use as a marker of preneoplastic oral lesions.

## Conclusion

The findings of this study indicate that BMI-1 expression increases in early oral carcinogenesis and that it may be associated with the occurrence of dysplastic changes. Furthermore, our findings indicate that both Ki-67 and BMI-1 are directly correlated and possibly play a role in initiation and progression of OSCC.
